# Novel PHL derivatives targeting quorum sensing: A strategy to combat *Pseudomonas aeruginosa* infections

**DOI:** 10.1016/j.bioflm.2025.100326

**Published:** 2025-10-24

**Authors:** Juanyan Liu, Yi Deng, Xinran Wang, Piao Luo, Shuaiwen Ding, Weizhong Zhang, Shahab Uddin, Hongyu Li, Yang Li

**Affiliations:** a Gansu High Throughput Screening and Creation Center for Health Products, School of Pharmacy, Lanzhou University, Lanzhou, China; b International Scientific and Technological Cooperation Base of Biopharmaceutical, School of Life Sciences, Institute of Microbiology, Lanzhou University, Lanzhou, China

**Keywords:** *Pseudomonas aeruginosa*, Quorum sensing, Antibiotic accelerant, Virulence, QSIs, Virtual screening

## Abstract

*Pseudomonas aeruginosa* (*P. aeruginosa*) is a common pathogenic bacterium that is widely distributed and highly pathogenic. Its pathogenicity is closely related to the formation of biofilms and virulence factors. It is well known that quorum sensing (QS) controls the formation of biofilms and the production of virulence factors in *P. aeruginosa*. Therefore, quorum-sensing inhibitors (QSIs) have attracted great interest as a potential therapeutic strategy against *P. aeruginosa* infections. This study departs from the conventional approach of screening potential QSIs through experimental synthesis and subsequent activity validation, which is typically time-consuming and labor-intensive. Instead, we integrated molecular docking, molecular dynamics simulations, and experimental synthesis with validation strategies to systematically evaluate newly designed Phenylacetanoyl homoserine lactones (PHLs) derivatives. In the computational screening phase, the binding affinity, binding sites, and binding stability between the PHL derivatives and the target protein LasR were comprehensively assessed using molecular docking and molecular dynamics simulations. Five compounds demonstrating promising potential QS inhibitory activity were selected. During the experimental phase, these five compounds underwent preliminary evaluation for their anti-biofilm activity against *P. aeruginosa*. Results indicated that N-(3-cyclobutyl lactone)-4-nitrobenzobutyramide (**L2**) exhibited the most potent biofilm inhibitory activity. Compared to previously reported QSIs, **L2** effectively inhibited biofilm formation, toxin production, and motility of *P. aeruginosa* at lower concentrations. Notably, **L2** exhibited characteristics of a partial agonist, which sheds light on its deeper mechanism of action. Furthermore, **L2** showed a synergistic effect when combined with antibiotics, significantly enhancing the susceptibility of biofilm bacteria of *P. aeruginosa* to antibiotics, while demonstrating a favorable hemocompatibility safety profile. The proposed combination strategy offers a new potential therapeutic avenue for the clinical treatment of *P. aeruginosa* infections.

## Highlights

• The quorum-sensing inhibitory activity of 24 PHLs derivatives against *Pseudomonas aeruginosa* was identified using molecular docking and dynamics simulations.
• Compared with other derivatives of PHLs, N-(3-cyclobutyl lactone)-4-nitrobenzobutyramide (**L2**) exhibits strong in vitro and in vivo activity against *Pseudomonas aeruginosa* at low concentrations.
• N-(3-cyclobutyl lactone)-4-nitrobenzobutyramide (**L2**) enhances the susceptibility of *Pseudomonas aeruginosa* biofilm bacteria to antibiotics, highlighting its potential for clinical applications.

## 1 Introduction

The pathogenicity of *Pseudomonas aeruginosa* (*P. aeruginosa*) was determined by its virulence factors and biofilm formation [1,2]. Virulence factors can damage host cells or evade clearance by the host immune system, ultimately leading to the development of disease. Biofilm formation enables bacteria to adhere to the host surface, protecting them from the host's immune responses and preventing antibiotics from penetrating and killing the bacteria within the biofilm [3]. Moreover, a concentration gradient of oxygen and nutrients exists within the biofilm, extending from the surface to the core. Bacteria on the surface are actively proliferating and metabolizing, while those in the deeper layers are dormant and exhibit tolerance to most antibiotics [4]. A substantial amount of research has demonstrated that quorum sensing (QS) significantly impacts the pathogenicity of *P. aeruginosa* by regulating its collective behavior, including biofilm formation, motility, and toxin production. Therefore, quorum-sensing inhibitors (QSIs) have garnered significant attention as a promising therapeutic strategy for combating *P. aeruginosa* infections.

In the study of QSIs against *P. aeruginosa*, traditional drug discovery approaches have primarily relied on structure-activity relationship (SAR) studies centered around natural autoinducer scaffolds and experimental-based screening methods [5]. This approach has led to the discovery of effective inhibitors, such as triazole-containing AHL analogues [5] and other synthetic ligands [6]. However, it is associated with significant limitations. Firstly, although the structure-activity relationship (SAR) approach can guide the design of novel compounds by analyzing the correlation between molecular structure and activity, it is limited by its reliance on finite experimental data and empirical hypotheses. This method is inherently inefficient, requiring the multi-step synthesis of a large number of analogues followed by biological activity screening, a process that is both time-consuming and costly. Secondly, and more critically, traditional SAR can be mechanistically opaque. It reveals which structures are active but often fails to elucidate the precise atom-level interactions within the receptor binding pocket that are responsible for binding [7,8].

In recent years, the advancement of computational methods and artificial intelligence (AI) technologies has positioned virtual screening (VS) and computer-aided drug design as emerging research priorities. Virtual screening utilizes techniques such as molecular docking and molecular dynamics simulations to provide a multidimensional assessment, thereby offering deeper insights into the dynamics, stability, and binding energy of inhibitor-receptor interactions—details that are largely unattainable through static SAR analysis. This approach enables more efficient screening of potential QSI candidates, significantly enhances screening throughput, and to some extent, mitigates the limitations associated with conventional methods [9]. In summary, while traditional SAR approaches offer certain guidance in QSI research, they present considerable limitations when addressing complex biological systems and high-throughput screening requirements. The introduction of virtual screening and computer-aided approaches provides novel perspectives and powerful tools for QSI investigation, offering a promising avenue for further advancing the development of QSI-based therapeutics against *P. aeruginosa*.

Geske and colleagues systematically investigated the structure-activity relationships of PHL-type QSIs by designing over 90 analogues [10,11]. Their work focused on key structural features, including acyl chain length, lactone stereochemistry, and the diversity of acyl functional groups. They observed that most of the PHL analogues exhibited inhibitory activity against the LasR protein, indicating substantial potential for the development of this QSI class. Furthermore, they highlighted that substituents on the phenyl ring—particularly electron-withdrawing groups at the para-position—significantly enhanced LasR inhibition. These findings provide valuable insights for the future structural optimization of PHL-based antagonists.

In this study, building upon the conventional paradigm for screening active compounds, we designed a focused compound library of 24 PHL-type QSIs based on the published structural characteristics of ligands targeting the LasR. This library was subsequently subjected to virtual screening, leading to the identification of five compounds with potential QS inhibitory activity. Among these, compound L2 demonstrated the most favorable binding affinity and structural stability throughout the screening process, suggesting its superior potential as a QS inhibitor. It is noteworthy that in subsequent experimental validation, compound L2 not only exhibited significant QS inhibitory activity but also surpassed our previously developed QSI by exerting stronger effects at lower concentrations, while also demonstrating characteristics of a partial agonist [10]. Furthermore, treatment with L2 significantly enhanced the susceptibility of *P. aeruginosa* biofilm bacteria to various antibiotics. The combination strategy proposed here offers a promising new direction for the clinical treatment of *P. aeruginosa* infections.

## 2 Materials and methods

### 2.1 Strains and media

*P. aeruginosa* PAO1 and *E. coli* OP50 were preserved in our laboratory. Clinical strains **C1**, **C2**, and **C3** of *P. aeruginosa* were provided by the Department of Clinical Laboratory, Gansu Provincial People's Hospital. These three clinical strains are all multidrug-resistant, and specific drug resistance information can be found in Supplementary Table S4. The reporter strains PAO1*-lasB-gfp*, PAO1-*rhlA-gfp*, and PAO1-*pqsA-gfp* were graciously provided by Professor Yang Liang from the School of Medicine, Southern University of Science and Technology. The *C. elegans* N2 strain was acquired from the Caenorhabditis Genetics Center (CGC). *P. aeruginosa* PAO1 was grown in Luria-Bertani (LB) medium at 37 °C and then streaked onto nutrient agar plates (Solarbio, Beijing, China) to obtain single colonies, which were stored at 4 °C. *C. elegans* was cultured on nematode growth medium (NGM) at 20 °C, and *E. coli* OP50 was used as the standard food source. **L1**, **L2**, **L6**, **L9**, **NO.10**, and **L10** were dissolved in dimethyl sulfoxide (DMSO) (Solarbio, Beijing, China).

### 2.2 Molecular docking

Molecular docking, a computational simulation technique used to predict the interactions between ligands and receptor active sites, has been established as a crucial tool for analyzing the interactions between compounds and their molecular targets [12]. In this study, molecular docking was used to simulate and analyze the binding ability of the 24 derivatives to the LasR protein of the Design of the PHLs derivatives library and in silico virtual screening. Firstly, the crystal structure of the LasR was obtained from the Protein Data Bank (PDB) database (http://www.rcsb.org/, PDB ID: 2UV0) [13]. The 2D structures of the derivatives were depicted using ChemDraw 17.0 software, and their 3D structures were built using Chem3D software. Finally, the file formats were converted using Open Babel software to standardize the file extensions to the PDB format. The derivatives were then visualized using PyMOL software.

Before molecular docking, the protein molecules and small-molecule ligands were preprocessed using PyMOL and AutoDockTools software. The water molecules in the protein structure were all eliminated. Hydrogen atoms were incorporated into the protein, and it was then selected as a receptor. Employing AutoDock software, 24 derivatives (Table 1) were chosen as ligands for molecular docking. The ligands were processed, including the addition of hydrogen atoms, adjustment of charges, determination of the ligand root, and identification of flexible torsions. Subsequently, the prepared ligands were saved in the PDBQT format for subsequent docking investigations. The docking site was defined based on the binding pocket of the LasR protein. Molecular docking was performed using AutoDock4 (version 4.2.6). The search parameters were set to a grid box of 60 Å × 60 Å × 60 Å, centered on the active site of the protein. The grid spacing was set to 0.375 Å, which is the default value in AutoDock4.

**Table 1:** Docking results of PHLs derivatives (Kcal/mol).

Compounds	Structure	OdDHL Energy	Compounds	Structure	Binding Energy
**L1**		−8.01	**L13**		−4.89
**L2**		−9.83	**L14**		−4.96
**L3**		−5.04	**L15**		−5.05
**L4**		+2.56	**L16**		−4.24
**L5**		−5.07	**L17**		−2.72
**L6**		−7.67	**L18**		−1.90
**L7**		−4.19	**L19**		−0.86
**L8**		−4.24	**L20**		−2.50
**L9**		−7.06	**L21**		−2.32
**L10**		−6.55	**L22**		−0.89
**L11**		−4.02	**L23**		−4.25
**L12**		−4.31	**L24**		−2.80
**Natural Ligand**		−7.74			

### 2.3 Molecular dynamics simulation

To further explore the interactions and stability between **LasR** and **L1, L2, L6, L9,** and **L10**, molecular dynamics (MD) simulations were conducted using GROMACS 2024.3 software. The protein-ligand complexes were simulated for a timescale of 100 ns. We generated the topology and force field files for the small molecules using Antechamber and for the protein using GROMACS. The AMBER14sb force field was used for the protein, the GAFF force field for the small molecules, and the TIP3P model for water. Molecular dynamics simulations were conducted using GROMACS 2024.3 at a temperature of 300 K.

### 2.4 Synthesis of PHLs derivatives

**Fig. 1:**
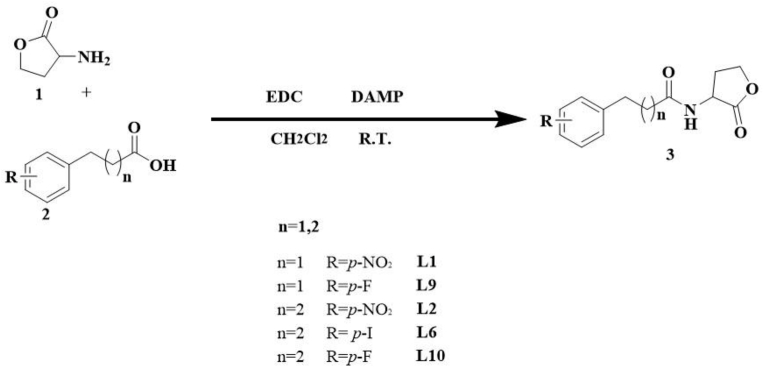
Synthesis of PHLs derivatives. RT = room temperature.

Based on molecular docking and dynamics results, **L1**, **L2**, **L6**, **L9**, and **L10** were synthesized for biological evaluation. The synthetic protocol described by Wei et al. [14] (Fig. 1), for detailed synthetic procedures, see supplementary materials.

### 2.5 Initial screening of derivatives

For the initial screening of the PHLs library, the standard strain PAO1 and clinical strains **C1**, **C2**, and **C3** of *P. aeruginosa* were used. Biofilm formation was assessed as the screening parameter, with **L1**, **L2**, **L6**, **L9**, and **L10** tested at concentrations ranging from 6.25 μM to 100 μM. The crystal violet assay, as described by Luo et al. [15], was employed to quantify biofilm formation. Each experiment was performed at least in triplicate.

### 2.6 Effects of L2 at various concentrations on the growth of *P. aeruginosa*

The growth of planktonic cells was determined following the method of Qu et al. [16]. *P. aeruginosa* was cultured overnight and subsequently diluted in an LB medium. The concentration of **L2** in the diluted bacterial suspension was adjusted to a range of 10–400 μM, followed by incubation at 37 °C. Samples of 1 ml were collected every 2 h, and the optical density at 600 nm (OD_600_) was measured using a spectrophotometer. A growth curve was then plotted based on the obtained data. Each experiment was performed at least in triplicate.

### 2.7 Observation of the inhibitory effect of L2 on the biofilm formation of *P. aeruginosa* by CLSM

This experimental approach, as described by Kumar et al. [17]. **L2** and **NO.10**, dissolved in DMSO, were added to the diluted *P. aeruginosa* PAO1 bacterial suspension to a final concentration of 25 μM. **NO.10** was used as the positive control, and an equivalent volume of DMSO served as the blank control. The fluorescence double-staining technique was adapted from the method of Kalia et al. [18]. After staining with fluorescent dyes, biofilm structural changes were assessed using CLSM. Each experiment was repeated at least three times. Imaging was performed using a Zeiss LSM 880 confocal laser scanning microscope system equipped with a 100 × Oil DIC objective. For the FITC-ConA dye, a 488 nm laser line was used for excitation, and emission was collected in the range of 500–550 nm. For the PI dye, a 561 nm laser line was applied for excitation, with emission gathered between 570 and 620 nm. All images were acquired under consistent parameters, including a pixel resolution of 1024 × 1024, laser power set at 2 %, and gain value fixed at 750 to ensure comparability across results. Image acquisition and subsequent processing were conducted using ZEN Blue software and ImageJ.

### 2.8 Evaluation of the inhibitory effect of L2 on the biofilm formation of *P. aeruginosa* observed by SEM

The inhibitory effect of **L2** on *P. aeruginosa* biofilm formation was evaluated using SEM, following the method of Luo et al. [15]. After bacterial treatment consistent with the CLSM biofilm structural analysis, samples were dehydrated with a graded ethanol series (10 min per step). The biofilm samples were air-dried in a desiccator for 24 h and then gold-suspended. **NO.10** was used as the positive control, and an equivalent volume of DMSO served as the blank control. Biofilm formation was observed and imaged using SEM. Each experiment was repeated at least three times. Sample morphology was observed using a Thermo Scientific Apreo S scanning electron microscope. Before imaging, the samples were sputter-coated with a 10 nm thick layer of platinum to enhance conductivity. Imaging was performed under the following conditions: an accelerating voltage of 25 kV, a working distance of 6 mm, and a secondary electron detector was used for signal acquisition.

### 2.9 Effect of L2 on the motility of *P. aeruginosa*

#### 2.9.1 Swimming

The swimming motility assay was performed based on the methods of Rashid and Kornberg et al. [19]. A swimming agar plate (comprising 10 g/L peptone, 5 g/L NaCl, and 0.3 % agarose) supplemented with **L2** was centrally inoculated with *P. aeruginosa* PAO1 using a sterile toothpick. **NO.10** served as the positive control, while an equivalent volume of DMSO was used as the blank control. After incubation at 30 °C for 12–14 h, the swimming motility area was quantified by measuring the diameter of the blue zone at the center of the plate. Each experiment was repeated at least three times.

#### 2.9.2 Swarming

The swarming assay method referred to the method of Kuma et al. [17]. The swarming plate consisted of nutrient agar (8 g/L) and glucose (5.0 g/L), with **L2** added at a concentration of 25 μM. **NO.10** was used as the positive control, and an equivalent volume of DMSO was included as the blank control. A 5 μL volume of overnight-cultured *P. aeruginosa* was inoculated onto the plate. After incubation at 30 °C for 18–24 h, the diameter of the swarming motility area at the plate's center was determined. Each experiment was repeated at least three times.

#### 2.9.3 Twitching

The twitching motility of *P. aeruginosa* PAO1 referred to the experimental method of Saqr et al. [20]. LB medium was employed in this study, with **L2** added at 25 μM. **NO.10** acted as the positive control, and an equivalent volume of DMSO served as the blank control. Overnight-cultured *P. aeruginosa* PAO1 was stabbed into the bottom of the culture dish. Following incubation at 37 °C for 48 h, the agar was removed, and the plate was air-dried and stained with crystal violet. After washing off the dye with sterile water, the twitching motility area was quantified. Each experiment was repeated at least three times.

### 2.10 Pyocyanin analyses

The inhibitory effect of **L2** on the production of pyocyanin of *P. aeruginosa* was evaluated by referring to the experimental method of O'Loughlin et al. [21]. A single colony of *P. aeruginosa* PAO1 was selected and aerobically cultured in a shaker at 37 °C for 24 h. The overnight culture was then transferred into an LB medium containing 25 μM of **L2** and incubated for an additional 24 h, after which the color of the culture was observed. **NO.10** was used as the positive control, and an equivalent volume of DMSO served as the blank control. Each experiment was repeated at least three times.

### 2.11 Elastase analyses

The effect of **L2** on the production of elastase was evaluated using the skim milk agar method of Hao et al. [22]. **NO.10** acted as the positive control, and an equivalent volume of DMSO was included as the blank control. The production of Elastase was quantified by measuring the diameter of the transparent zone on the plate. Each experiment was repeated at least three times.

### 2.12 Effect on the expression of QS-regulated genes

The expression levels of key QS-regulated genes (*lasI, lasR, rhlI, rhlR, mvfR*) in *P. aeruginosa* PAO1 were measured in the presence of **L2**. **NO.10** was the positive control, and 16S rRNA was the internal reference. Primers are provided in Table S3 (Supplementary Materials). Total RNA was isolated using the Ultra-pure RNA Kit (CWBIO, Beijing, China), and cDNA was synthesized using Hifair® III 1st Strand cDNA Synthesis SuperMix for qPCR (Yeasen, Beijing, China). qPCR was conducted using Hieff UNICON® Universal Blue qPCR SYBR Green Premix (Yeasen, Beijing, China), and gene expression was analyzed using the 2^−ΔΔCt^ method. All experiments were performed in triplicate for technical and biological repetition.

### 2.13 Inhibition of *GFP* reporter strains

This experimental method was based on the protocol of Zhang et al. [23]. The overnight-cultured *P. aeruginosa* PAO1*-lasB-gfp* was diluted with fresh medium to an OD_600_ of 0.05. The **L2** was adjusted to a final concentration of 25 μM in a 96-well plate. **NO.10** acted as the positive control, and an equivalent volume of DMSO was used as the blank control. The microtiter plate was incubated at 37 °C in a Multimode Plate Reader to measure the cell density (OD_600_) and *GFP* fluorescence (excitation at 485 nm, and emission at 535 nm) at 15 min intervals for at least 16 h. A similar method was performed for the inhibition of *P. aeruginosa* PAO1-*rhlA-gfp* and PAO1-*pqsA-gfp*. All experiments were performed in triplicate for technical and biological repetition.

### 2.14 Determination of the MIC and in vitro activity in combination with antibiotics

The determination of the MIC was carried out following the experimental method described by Liu et al. [23]. The experimental method for evaluating the inhibitory effect of the combination of antibiotics and **L2** on the growth of bacteria within the PAO1 biofilm referred to the experimental approach of Maura and Rahme [24]. The concentration of antibiotics was all selected to be half of the MIC of *Pseudomonas aeruginosa* biofilm bacteria. A mature biofilm of *P. aeruginosa* PAO1 was formed on a coverslip and rinsed twice with 1 × PBS to remove planktonic bacteria. **L2** (1.56 μM–200 μM) was combined with amikacin (2.5 μg/mL) in LB medium, with six replicate wells per group. Control groups included amikacin or **L2** alone. The 96-well plate was incubated statically at 37 °C for 18–20 h, and the OD_600_ was measured to assess the combined effect on biofilm bacterial growth. The same protocol was used to evaluate combinations of **L2** with ceftazidime (20 μg/mL), carbenicillin (2.5 μg/mL), and trimethoprim (5 μg/mL). All experiments were performed in triplicate for technical and biological repetition.

### 2.15 CLSM analysis of the combined effects of amikacin and L2 on the biofilm structure of *P. aeruginosa*

This study followed the method of Kumar et al. [25]. **L2** and **NO.10** were added to the diluted PAO1 bacterial solution at 25 μM, with amikacin at 2.5 μg/mL. **NO.10** was the positive control, and an equivalent volume of DMSO served as the blank control. One milliliter of the treated bacterial solution was added to each well containing a glass slide, with three replicates per group. After 24 h of static incubation at 37 °C, the wells were rinsed three times with PBS to remove planktonic bacteria. The fluorescence double-staining method, based on Kalia et al. [26], was then applied. Each experiment was repeated at least three times. Imaging was performed using a Zeiss LSM 880 confocal laser scanning microscope system equipped with a 100 × Oil DIC objective. For the FITC-ConA dye, a 488 nm laser line was used for excitation, and emission was collected in the range of 500–550 nm. For the PI dye, a 561 nm laser line was applied for excitation, with emission gathered between 570 and 620 nm. All images were acquired under consistent parameters, including a pixel resolution of 1024 × 1024, laser power set at 2 %, and gain value fixed at 750 to ensure comparability across results. Image acquisition and subsequent processing were conducted using ZEN Blue software and ImageJ.

### 2.16 *C. elegans* fast-killing assay

The *C. elegans* fast-killing assay was used in this experiment, adapted from the methods of Cezairliyan and O'Loughlin et al. [21,27], to evaluate the anti-*P. aeruginosa* infection efficacy of **L2**. **L2** was added to *P. aeruginosa* PAO1 at 25 μM and cultured at 37 °C for 24 h. **NO.10** acted as the positive control, and an equivalent volume of DMSO was used as the blank control. The treated PAO1 culture was inoculated onto PGS agar plates (1 % peptone, 1 % NaCl, 1 % glucose, 150 mM sorbitol, and 1.7 % agar) and incubated at 37 °C for 24 h. Synchronized L4-stage *C. elegans* (25–30 nematodes per plate) were added to the plates, and survival was recorded every 3 h at 25 °C. All experiments were performed in triplicate for technical and biological repetition.

### 2.17 Hemolysis assay

This experiment followed the method of Lin et al. [28]. Freshly isolated mouse red blood cells were washed three times with PBS buffer to prepare a 5 % suspension. **L2** was diluted in PBS buffer to concentrations of 12.5 μM, 25 μM, 50 μM, and 100 μM in a 200 μL system. The compound was mixed with the red blood cell suspension and incubated at 37 °C for 1 h. After centrifugation at 1500 rpm for 10 min, 30 μL of the supernatant was transferred to a 96-well plate containing 70 μL of PBS buffer. Absorbance at 540 nm was recorded using a microplate reader. PBS buffer was the negative control, and 2 % Triton X-100 was the positive control. All experiments were performed in triplicate for technical and biological repetition.

## 3 Results

### 3.1 Compound design, virtual screening, and synthesis

#### 3.1.1 Design strategy

Based on the established structure-activity relationship (SAR) of PHL-type QSI, this study retained the lactone and benzene ring as the core framework and modified the amide side chain length and benzene ring substituents (position, type, and quantity) of PHL-type QSI. As a result, a PHL library containing 24 different derivatives was successfully established (Table 1).

#### 3.1.2 Molecular docking

**Fig. 2:**
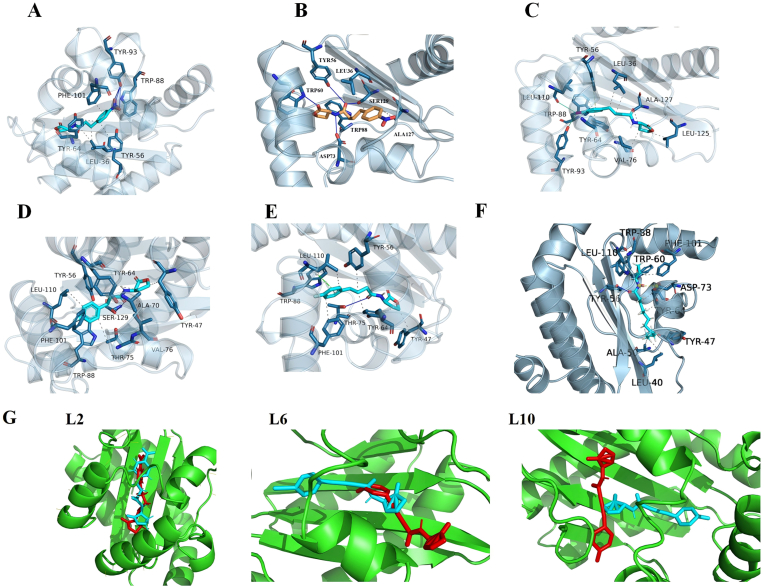
(A) Analysis of the Interactions between LasR and L1. (B) Analysis of the Interactions between LasR and L2. (C) Analysis of the Interactions between LasR and L6. (D) Analysis of the Interactions between LasR and L9. (E) Analysis of the Interactions between LasR and L10. (F) Analysis of the Interactions between LasR and natural ligand. The blue solid lines represent hydrogen bonds, the gray dashed lines represent hydrophobic interactions, the green dashed lines represent *π*-stacking, and the cyan solid lines represent halogen bonds. (G) The conformation of L2, L6, and L10 at 0ns and 100ns. Blue color indicates the conformation at 0ns, red color indicates the conformation at 100 ns.

Binding energies of the compounds to the LasR were calculated using AutoDock4 scoring functions, with lower values indicating stronger ligand-protein binding affinity [29]. Studies have shown that a binding energy below −5.5 kcal/mol suggests a moderate to strong binding affinity between the receptor and ligand [26,[30], [31], [32]]. The docking results were summarized in Table 1, and **L1**, **L2**, **L6**, **L9**, and **L10** with lower binding energies (8.01, −9.83, −7.67, −7.06, and −6.55 kcal/mol, respectively) were selected for further evaluation. Studies have indicated that lower binding energies are generally associated with stronger QS inhibitory activities [3]. To further identify the binding sites of **L1**, **L2**, **L6**, **L9**, natural ligand, and **L10** on LasR, protein-ligand interaction analysis was performed. The results were illustrated in Fig. 2A–F.

The nitro group on the benzene ring of **L1** formed three hydrogen bonds with Tyr93 within the binding pocket and engages in hydrophobic interactions with amino acids, including Tyr56, Trp88, Phe101, and Leu36. **L2** formed hydrogen bonds with Try56, Trp60, Asp73, and Ser129, as well as hydrophobic interactions with amino acids such as Trp88, Ala127, and Leu36. **L6** showed a π-bond with Tyr56, halogen bonds with Tyr93 and Leu110, and hydrophobic interactions with amino acids such as Val76, Leu125, and Ala127. **L9** formed a hydrogen bond with Ser129 and hydrophobic interactions with amino acids, including Trp88, Phe101, Val76, and Tyr56. **L10** forms a π-bond with Trp88, a hydrogen bond with Thr75, and hydrophobic interactions with amino acids such as Leu110. As shown in Fig. 2B and F, we also found that the interaction between **L2** and LasR receptors by formation of H-bonds with the Try 56, Trp 60, and Asp 73 is consistent with the binding site of the natural ligand (OdDHL) [25].

#### 3.1.3 Molecular dynamics simulation

**Fig. 3:**
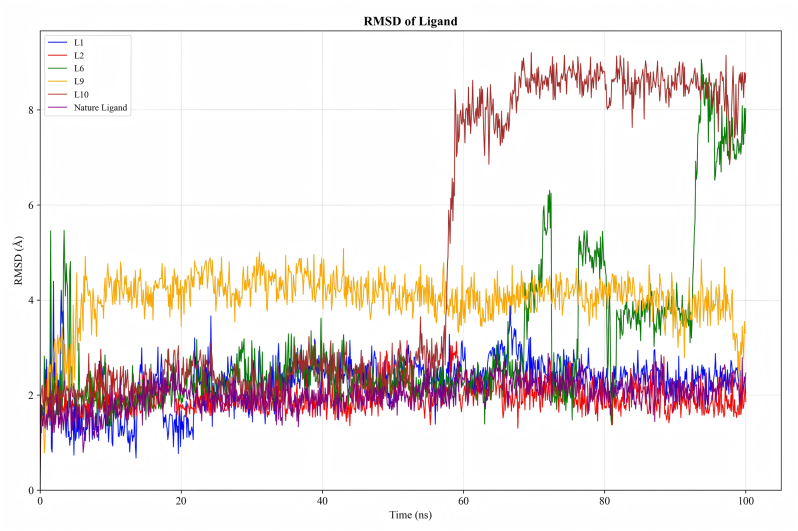
RMSD dynamics of L1, L2, L6, L9, L10, and natural ligand within the LasR protein pocket.

The RMSD values of the ligands are shown in Fig. 3. **L1** stabilized at 2.0–2.5 Å after 20 ns of MD simulation. **L2** was highly stable; the purple curve represents the RMSD of the natural ligand. It can be seen that the RMSD values of compound L2 highly coincide with that of the natural ligand, with an RMSD of 1.5–2.0 Å. As shown in Fig. 2G, comparison of the conformations of **L2** at 0 ns and 100 ns revealed minimal conformational changes after 100 ns of simulation, consistent with the results of molecular dynamics simulations. **L9** stabilized at 4.0–4.5 Å after 8 ns. In contrast, **L6** and **L10** exhibited significant fluctuations after 60 ns. Analysis of conformations at 0 ns and 100 ns revealed substantial changes in **L6** and **L10**; the altered conformations are shown in Fig. 2G. In conclusion, among the 24 compounds we designed, molecular docking results indicated that compounds L1, L2, L6, L9, and L10 exhibited high binding affinity to the receptor protein, leading to our initial prediction that these five compounds might possess good QS inhibitory activity. However, since the biological activity of a compound is influenced not only by binding affinity but also by the stability of the ligand–receptor interaction—a factor that molecular docking alone cannot fully capture—we further employed molecular dynamics (MD) simulations to assess the binding stability of these five candidate compounds. The MD simulation results revealed that although L1, L6, L9, and L10 displayed relatively high binding affinity, their root-mean-square deviation (RMSD) values during the binding process were higher and exhibited greater fluctuation compared to those of L2. This suggests that among the five candidates, L2 formed the most stable complex with the receptor protein throughout the simulation. Therefore, we further concluded that L2 is expected to exhibit the best QS inhibitory activity among the five compounds.

#### 3.1.4 Synthesis of PHLs derivatives

The synthesized derivatives were characterized by NMR, confirming purities of ≥95 %. Detailed NMR spectra are provided in Supplementary Table S1.

### 3.2 In vitro activity evaluation of compound L2 as monotherapy

#### 3.2.1 Initial screening of derivatives

The inhibitory activities of **L1**, **L2**, **L6**, **L9**, and **L10** against biofilm formation by standard strain PAO1 and clinical strains **C1**, **C2**, and **C3** were evaluated at various concentrations. As shown in Table 2, **L1** demonstrated the lowest inhibition rate against PAO1, yet exhibited a highly significant activity against the clinical strain **C3**. **L6** displayed a superior inhibitory effect on the clinical strain **C2** but showed poor performance against the other three strains. **L9** demonstrated comparatively lower inhibitory efficacy in preventing biofilm formation across all tested strains. **L10** had a notable inhibitory effect on the clinical strain **C3**, although it performed inadequately against the remaining three strains**.** Notably, **L2** exhibited significantly inhibitory effects against both the standard and clinical strains of *P. aeruginosa* than **L1**, **L6**, **L9**, **L10**, and **NO.10**. Furthermore, **L2** demonstrated its strongest inhibitory activity against biofilm formation at 25 μM. Based on the statistical analysis, compound L2 exhibited the most potent inhibition of biofilm formation against all four tested strains of *P. aeruginosa* compared to the other compounds. Since the inhibitory effect was most significant at 25 μM, L2 at this concentration was selected for subsequent evaluation.

**Table 2:**
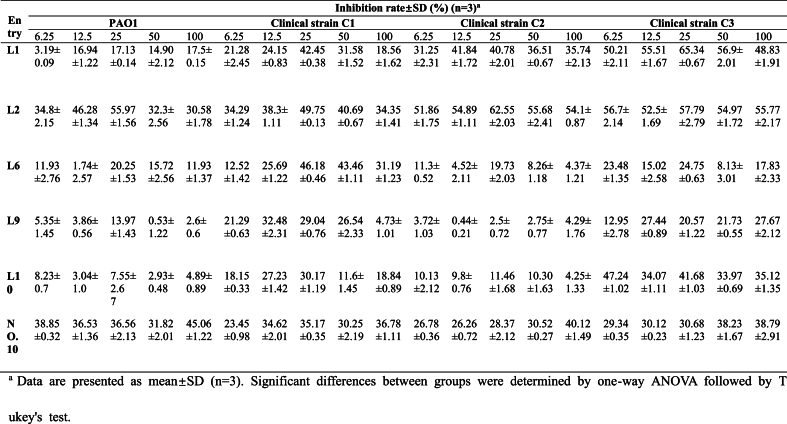
Biofilm inhibition of compound **L1-NO.10** across four *P. aeruginosa* strains (6.25–100 μM).

#### 3.2.2 Confocal laser scanning microscopy (CLSM) imaging of biofilm treated with L2

**Fig. 4:**
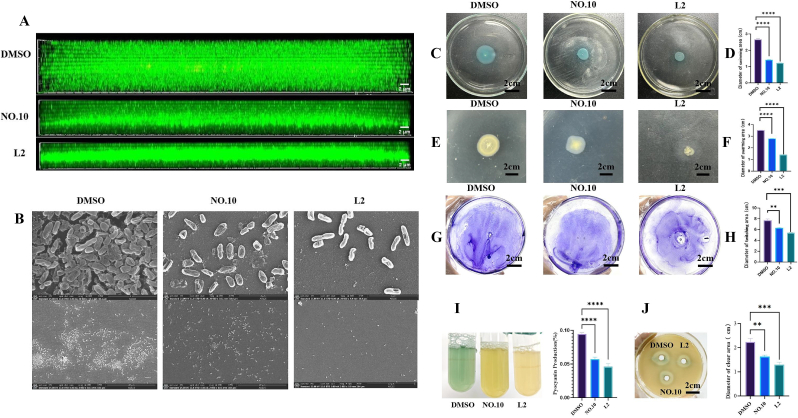
(A) CLSM imaging of biofilm treated with L2. (B) SEM of biofilm morphology of *P. aeruginosa* PAO1. The three pictures above were captured at a magnification of X20000, while the three pictures below were captured at a magnification of X2000. (C) (D) represents swimming motility. (E) (F) represents swarming motility. (G) (H) represents twitching motility. (I) Pyocyanin analyses. (J)Elastase analyses. Data are presented as the mean ± standard error of the mean of at least three independent biological replicates (n = 3); Graphs were generated using GraphPad Prism software (V.10.0). Statistical significance was determined by one-way ANOVA performed in SPSS (V.23.0). *P*-values less than 0.05 were considered statistically significant. Significance levels are denoted as follows: ns, not significant (*p* > 0.05); ∗*p* < 0.05,∗∗*p* < 0.01,∗∗∗*p* < 0.001,∗∗∗∗*p* < 0.0001.

To further illustrate the effects of **L2** on *P. aeruginosa* PAO1 biofilm formation, biofilm structural morphology was visualized by CLSM. In this method, green fluorescence indicated biofilm thickness, while red fluorescence correlated with the number of dead cells. As shown in Fig. 4A, no red fluorescence was detected in the **L2**-treated groups, suggesting that **L2** did not cause bacterial cell death, which is consistent with the results of the growth inhibition effect of **L2** against PAO1 (Supplementary Fig. S1). CLSM analysis revealed that the biofilm thickness in the control group was about 18 μm. In contrast, the biofilm thickness in the **NO.10** and **L2** treatment groups decreased to nearly 8 μm and 5 μm, respectively. These results indicated that **L2** exhibited significantly inhibitory activity against biofilm formation by PAO1.

#### 3.2.3 Biofilm morphology under a scanning electron microscope (SEM)

The impact of **L2** on *P. aeruginosa* biofilm formation was further assessed using SEM. As shown in Fig. 4B, the control group exhibited a dense, multilayered biofilm with tightly packed bacterial cells. In contrast, the biofilms in the treated groups were thinner, less uniform, and more loosely structured, with a less dense bacterial distribution. These results indicated that both **L2** and **NO.10** significantly reduced biofilm formation. Furthermore, the biofilm structure in the **L2**-treated group was more loosely organized compared to that in the **NO.10**-treated group.

#### 3.2.4 Effect of L2 on the motility of *P. aeruginosa*

The motility of *P. aeruginosa* is closely related to the formation of biofilm. Therefore, we investigated the effect of **L2** on the motility of *P. aeruginosa*. As shown in Fig. 4C–H, the swimming, swarming, and twitching motility ranges of *P. aeruginosa* PAO1 in the control group were 2.70 ± 0.06 cm, 3.50 ± 0.15 cm, and 8.00 ± 0.12 cm, respectively. In the presence of **NO.10**, these ranges were reduced to 1.40 ± 0.04 cm, 2.80 ± 0.12 cm, and 6.40 ± 0.18 cm, respectively. In contrast, in the presence of **L2**, the swimming, swarming, and twitching motility ranges were significantly reduced to 1.20 ± 0.05 cm, 1.40 ± 0.11 cm, and 5.50 ± 0.06 cm, respectively. Compared to **NO.10**, the inhibition rates were increased by 14.29 ± 0.35 %, 50.00 ± 0.57 %, and 14.06 ± 1.32 %, respectively. Overall, **NO.10** and **L2** significantly inhibited the motility of PAO1, and the inhibitory activity of L2 was stronger than that of NO.10.

#### 3.2.5 Pyocyanin analyses

Pyocyanin is a green exotoxin generated by *P. aeruginosa* governed by the QS system. As shown in Fig. 4I, both **NO.10** and **L2** inhibited pyocyanin production by PAO1. Compared to the control group, **NO.10** reduced pyocyanin production by 39.55 ± 0.23 %, while **L2** reduced it by 51.12 ± 0.21 %. Thus, **L2** demonstrated a significantly inhibitory effect on pyocyanin production compared to **NO.10**.

#### 3.2.6 Elastase analyses

*P. aeruginosa* produces elastase, which facilitates pathogen adhesion and causes damage to host tissues and vascular invasion. As shown in Fig. 4J, both **NO.10** and **L2** inhibited the production of elastase. Compared to the control group, the clear zone diameter on skim milk agar decreased by 26 ± 1.02 % with **NO.10** and by 41 ± 0.87 % with **L2**, indicating that **L2** exhibited a greater inhibitory effect on elastase production than **NO.10**.

#### 3.2.7 Effect on expression of QS-regulated genes

**Fig. 5:**
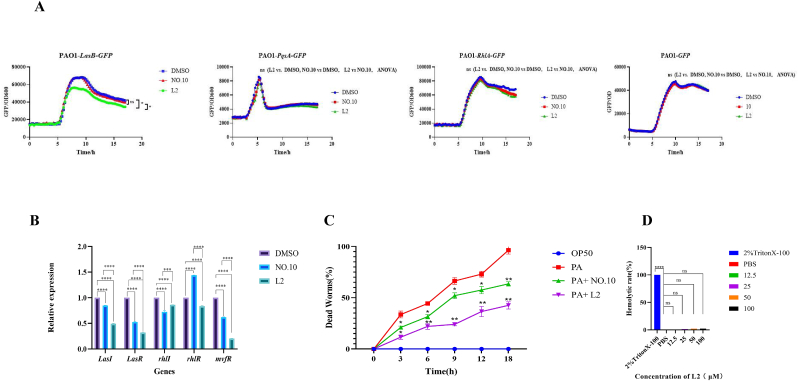
(A) Inhibition of *GFP* reporter strains. (B) Effect on expression of QS-regulated genes. (C) In vivo *C. elegans* survival assay, the PA + L2 and PA + NO.10 groups were compared with the PA group. (D) The hemolysis rates of mouse red blood cells induced by L2 at different concentrations. 2 % Triton X-100 was used as a positive control, and PBS was used as the negative control. Data are presented as the mean ± standard error of the mean of at least three independent biological replicates (n = 3); Graphs were generated using GraphPad Prism software (V.10.0). Statistical significance was determined by one-way ANOVA performed in SPSS (V.23.0). *P*-values less than 0.05 were considered statistically significant. Significance levels are denoted as follows: ns, not significant (*p* > 0.05); ∗*P* < 0.05, ∗∗*P* < 0.01, ∗∗∗*P* < 0.001, ∗∗∗∗*P* < 0.0001.

To further determine the inhibition mechanisms of **L2** on virulence and biofilm formation in *P. aeruginosa* PAO1, the expression levels of QS-regulated Genes were assessed using qPCR. As shown in Fig. 5B, the relative expression levels of QS-related genes *lasI* (16 ± 0.76 %), *lasR* (47 ± 2.01 %), *rhlI* (25 ± 0.39 %), and *mvfR* (38 ± 0.83 %) were observed to be decreased in the **NO.10** treatment group. However, upregulated relative expression levels of *lasI* were observed. In contrast, **L2** inhibited the expression of all five QS genes, with relative expression levels of *lasI, lasR, rhlI, rhlR,* and *mvfR* decreasing by 48 ± 0.34 %, 68 ± 0.62 %, 16 ± 0.28 %, 17 ± 1.01 %, and 79 ± 0.36 %, respectively. Importantly, the inhibition activity of **L2** on the target pathway genes *lasI* and *lasR* was significantly higher than that of **NO.10**.

#### 3.2.8 Inhibition of *GFP* reporter strains

Three *GFP* reporter strains, PAO1-*lasB*-*gfp*, PAO1-*rhlA*-*gfp*, and PAO1-*pqsA*-*gfp*, were used to further validate the effect of **L2** on three different QS systems in *P. aeruginosa*. As shown in Fig. 5A, **L2** significantly inhibited the fluorescence expression of PAO1-*lasB-gfp* and PAO1-*rhlA-gfp*. In contrast, **NO.10** only mildly inhibited the fluorescence expression in both reporter strains. These results indicated that both **L2** and **NO.10** can directly target the LasB and RhlA QS systems of *P. aeruginosa*. However, **L2** exhibited a stronger inhibitory effect than **NO.10**, which is consistent with previous results.

### 3.3 In vivo activity assessment of compound L2 monotherapy using the *Caenorhabditis elegans* (*C. elegans)* model

The protective effects of L2 against *P. aeruginosa* were examined using *C. elegans* as an animal model. As shown in Fig. 5C, compared with the blank control group, the *C. elegans* mortality rate was significantly reduced by both L2 and NO.10. Within 6 h of incubation, the survival rate of *C. elegans* in the L2 group was nearly 80 % and remained at 60 % after 18 h. In contrast, the survival rate in the NO.10 group was approximately 60 % at 6 h and decreased to 40 % after 18 h. Overall, *C. elegans* was effectively protected from the lethal effects of *P. aeruginosa* PAO1 by L2, which exhibited a significantly protective effect than **NO.10.**

### 3.4 Synergistic effects with antibiotics

#### 3.4.1 *In vitro* activity in combination with antibiotics

**Fig. 6:**
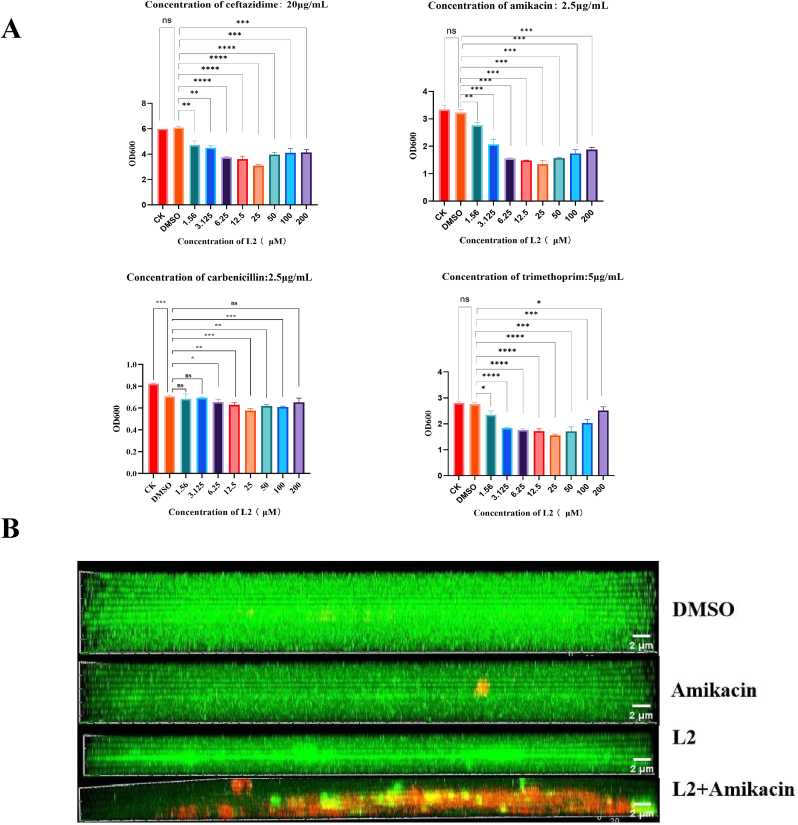
(A) In vitro activity of L2 in combination with antibiotics. (B) CLSM imaging of *P. aeruginosa* biofilm after amikacin and L2 combination treatment. Data are presented as the mean ± standard error of the mean of at least three independent biological replicates (n = 3); Graphs were generated using GraphPad Prism software (V.10.0). Statistical significance was determined by one-way ANOVA performed in SPSS (V.23.0). *P*-values less than 0.05 were considered statistically significant. Significance levels are denoted as follows: ns, not significant (*p* > 0.05); ∗*p* < 0.05; ∗∗*p* < 0.01; ∗∗∗*p* < 0.001; ∗∗∗∗*p* < 0.0001.

As shown in Supplementary Table S2, the MIC of ceftazidime against *P. aeruginosa* biofilm bacteria was 32 times higher than that against planktonic bacteria. Similarly, the MICs of carbenicillin and trimethoprim against PAO1 biofilm bacteria were 2-and 8 times higher, respectively. It was hypothesized that **L2** enhances the antibiotic susceptibility of *P. aeruginosa* biofilm bacteria by inhibiting biofilm formation. The inhibitory effects of sub-inhibitory concentrations of antibiotics in combination with **L2** on *P. aeruginosa* biofilm bacteria growth are shown in Fig. 6A. L2 enhanced the inhibitory effect of antibiotics on *P. aeruginosa* biofilm bacteria in a concentration-dependent manner. The most significant enhancement was observed at 25 μM of **L2**. Specifically, at this concentration, biofilm bacterial growth was inhibited by 48 ± 0.24 %, 60 ± 0.52 %, 30 ± 0.34 %, and 45 ± 1.82 % for ceftazidime, amikacin, carbenicillin, and trimethoprim, respectively. To evaluate the potential of L2 as an antibiotic adjuvant, we assessed its combination with four antibiotics against *P. aeruginosa*. The Fractional Inhibitory Concentration Index (FICI) analysis revealed a strongly synergistic interaction between L2 and three of the antibiotics (ceftazidime, amikacin, and trimethoprim) across the entire tested concentration range of L2 (12.5–200 μM). Synergy with the fourth antibiotic (carbenicillin) was demonstrated when L2 was applied at concentrations of 12.5–50 μM. In summary, a significant synergistic effect was observed between L2 and the tested antibiotics. L2 effectively potentiated the efficacy of antibiotics and markedly increased the susceptibility of *P. aeruginosa* biofilm bacteria. It is noteworthy that the combination of L2 with amikacin exhibited the strongest inhibitory activity against the biofilm cells.

#### 3.4.2 CLSM imaging of *P. aeruginosa* biofilm after amikacin and L2 combination treatment

Combination therapy with antibiotics may achieve superior therapeutic effects, whereas QSIs alone may not eradicate bacterial infections, especially in immunocompromised individuals. The impact of amikacin and **L2** on PAO1 biofilms was investigated using a fluorescence dual-staining method adapted from Kalia et al. [26]. *P. aeruginosa* biofilms from different treatment groups were stained with FITC-Con A and PI and observed under CLSM. As shown in Fig. 6B, biofilm thickness was nearly 12 μm in the control group. It was reduced to nearly 9 μm after amikacin treatment, and weak red fluorescence was observed, indicating a limited bactericidal effect against biofilm bacteria of *P. aeruginosa* PAO1. In contrast, the biofilm thickness was reduced by nearly 6 μm in the **L2**-treated group with no red fluorescence, indicating that it could inhibit biofilm formation without killing the bacteria. Interestingly, the biofilm thickness was reduced to 5 μm, and red fluorescence was significantly increased when **L2** was combined with amikacin as compared to the other groups. These results demonstrated that the **L2**-amikacin combination exhibited significant bactericidal effects against PAO1 biofilm bacteria. In general, L2 enhanced the sensitivity of PAO1 biofilm bacteria to amikacin, and the inhibition of biofilm formation by the L2-amikacin combination was more obvious.

### 3.5 Hemolysis assay

To determine whether **L2** was toxic to mammalian cells, a hemolysis assay was performed with mouse red blood cells. Triton X-100, a nonionic surfactant, was used as a positive control, the hemolysis rate of which was set to 100 %. As shown in Fig. 5D, the hemolysis rates of **L2** at concentrations of 12.5, 25, 50, and 100 μM were 0.05 %, 1.5 %, 2.1 %, and 2.3 %, respectively. Even at the highest concentration of 100 μM, the hemolysis rate remained below 2.5 %, indicating that **L2** had little hemolytic effect against mouse red blood cells.

## 4 Discussion

The QS system has been shown to regulate a variety of physiological activities of *P. aeruginosa*, including biofilm formation, production of virulence factors, and bacterial motility. It is one of the key regulatory mechanisms underlying the pathogenicity of *P. aeruginosa* [33]. Among the four QS systems in *P. aeruginosa*, the LasI/LasR system is hierarchical at the top level. Therefore, LasR has been targeted in numerous studies to design different types of non-natural QSIs to inhibit the QS system of *P. aeruginosa* [[34], [35], [36]]. Traditional approaches to developing quorum sensing inhibitors (QSIs) against *Pseudomonas aeruginosa* have largely depended on structure-activity relationship (SAR) studies derived from natural autoinducer scaffolds, complemented by experimental screening. Although these methods have yielded some effective inhibitors, they suffer from inherent drawbacks, including low efficiency, high cost, and limited mechanistic insight into binding interactions at the atomic level. In recent years, virtual screening (VS) and computer-aided drug design have emerged as powerful alternatives. By employing molecular docking and dynamics simulations, VS provides multidimensional analysis of inhibitor-receptor interactions—such as binding stability, dynamics, and free energy—that are difficult to capture with conventional SAR. This computational approach significantly enhances screening efficiency and throughput, while also offering deeper mechanistic elucidation, thereby overcoming major limitations of traditional strategies and accelerating the development of novel QSI-based therapeutics.

Based on the PHLs SAR summarized in previous studies, a compound library composed of 24 PHL derivatives was designed. Molecular docking and dynamics simulations were then performed on the library. The docking results revealed that **L1**, **L2**, **L6**, **L9**, and **L10** exhibited lower binding energies than the other derivatives. Generally, derivatives with lower binding energies tend to have stronger QS inhibitory activity [4]. Previous studies have shown that QSIs have stronger inhibitory effects when their binding sites on LasR were similar to those of the natural ligand [37,38]. In this study, the binding energy of L2 was the lowest, and the key amino acid binding sites of L2 to the receptor were consistent with those of the natural ligand (OdDHL) [25]. Dynamics simulations revealed that **L2** exhibited the most stable docking conformation compared to the other four compounds. Based on these results, it is predicted that **L2** has more prominent QS inhibitory activity.

To further validate the in-silico studies, **L1**, **L2**, **L6**, **L9**, and **L10** were synthesized, and their inhibitory activities against biofilm formation by *P. aeruginosa* were assessed. The results showed that compared to **L1**, **L6**, **L9**, **L10**, and **NO. 10**, **L2** exhibited stronger inhibitory activity against biofilm formation by the PAO1. As shown in Table 2, we observed a notable phenomenon wherein compound L2 exhibited maximal inhibitory activity at an intermediate concentration (25 μM), with diminished effects at higher concentrations (50–100 μM). This non-monotonic dose–response curve is consistent with the established concept of “partial agonism” within the field of quorum sensing inhibitor (QSI) research [10]. A partial agonist is a ligand that binds to the receptor yet does not elicit a full transcriptional response, even under conditions of complete receptor occupancy. At low concentrations, such compounds primarily function as competitive antagonists by occupying the ligand-binding pocket and preventing access of the native, high-efficacy autoinducer (e.g., OdDHL). This effectively silences the quorum sensing pathway, accounting for the potent inhibition we observed at 25 μM. However, when the concentration of the partial agonist is substantially increased, the absolute number of ligand–receptor complexes formed becomes sufficient to trigger a low level of transcriptional activation. This intrinsic agonistic activity thereby attenuates the net inhibitory effect observed at higher concentrations [10,39]. This model provides a compelling mechanistic explanation for the attenuated efficacy of L2 observed at 50 and 100 μM. A similar phenomenon has been reported by Geske et al., who further analyzed the underlying reasons for its occurrence [10]. Certain synthetic AHL analogues function as potent antagonists of *V. fischeri* LuxR at low concentrations but transition into agonists at higher concentrations (>100 μM), and are therefore defined as partial agonists. The behavior of L2 aligns with this pattern, indicating that it acts as a partial agonist of LasR.

This study observed that the predicted trends of compound activity by molecular docking and molecular dynamics simulation were consistent with the results of in vitro anti-biofilm activity experiments. Specifically, although compounds L1, L6, L9, and L10 all demonstrated favorable initial binding affinity to the LasR receptor in molecular docking simulations, their inhibitory potency was significantly lower than that of L2 and exhibited notable strain specificity. This phenomenon was rationally explained by subsequent molecular dynamics (MD) simulations. As a static modeling approach, molecular docking successfully identified compounds with high initial binding potential; however, it could not adequately assess the dynamic stability of the complexes over time. Critical MD trajectory analyses revealed that only the L2–LasR complex maintained the lowest and most stable RMSD values throughout the simulation, indicating a robust and persistent binding pose. Conversely, the complexes of L1, L6, L9, and L10 exhibited elevated RMSD values with significant fluctuations, indicating an unstable binding mode where the ligands undergo translocation or conformational rearrangement within the binding pocket. This dynamic instability reduces their effective binding affinity and serves as the primary reason for their weaker activity in biological assays. Furthermore, for compounds such as L1 and L10, which exhibit notable inhibitory activity only against specific clinical strains, we hypothesize that their unstable binding modes make their efficacy more susceptible to minor amino acid variations or conformational flexibility in the LasR protein across different strains. In contrast, owing to its stable binding, the inhibitory activity of L2 is less affected by such genetic background differences, thereby demonstrating broad-spectrum and consistent inhibitory efficacy.

Molecular docking revealed distinct binding sites between L2 and reported LasR inhibitors. Specifically, L2 formed hydrogen bonds with TYR56, TRP60, ASP73, and SER129. In contrast, the known inhibitors interacted primarily through different residues: methyl gallate (MG) formed three hydrogen bonds with TRP60, ARG61, and THR75 [40]; metformin established four hydrogen bonds with ASP73, THR75, THR115, and SER129 [25]; the C30 furanone, a known LasR inhibitor, bound to TRP60 and ARG61 via hydrogen bonds [41]; and sitagliptin interacted with LasR by forming a hydrogen bond with TRP60 [42].

A key distinction exists in the binding sites to the target LasR between known QSIs such as MG and metformin, and the compound L2. While MG and metformin form hydrogen bonds with only one or two key amino acid residues of the receptor, L2 mimics the native ligand by engaging four critical residues via hydrogen bonding. This difference in binding interactions directly accounts for the marked divergence in their biological activities. Specifically, MG achieved a 68.1 % inhibition of pyocyanin production in *Pseudomonas aeruginosa* PAO1 at a concentration as high as 256 μg/mL, and metformin showed only 48.67 % inhibition at 10 mg/mL. In contrast, L2 reached an inhibition rate of 51.12 % at a much lower concentration of just 25 μM (approximately 7.3 μg/mL). These results clearly demonstrate that the superior binding site of L2 enables highly efficient suppression of virulence factor production at substantially lower concentrations.

To further elucidate the inhibitory mechanism of **L2** on the biofilm formation and virulence factors of *P. aeruginosa*, we employed qPCR to assess the expression levels of key QS-related genes. Compared to the previously published QSI derivative (NO. 10), L2 exhibited a more pronounced down-regulation of the five genes: *LasI, LasR, rhlI, rhIR,* and *mvfR*. **L2** targets the LasR protein, leading to the downregulation of *lasI*, *lasR, rhlI, and rhlR*. Furthermore, *mvfR,* which is crucial for the production of pyocyanin and elastase, is positively regulated by the Las pathway [43,44], and the downregulation of *mvfR* significantly contributes to inhibiting the production of pyocyanin and elastase.

The integration of QS inhibitors with antibiotics has been recognized as a prominent therapeutic avenue for alleviating infection severity [45]. Our experimental results showed that the susceptibility of *P. aeruginosa* to ceftazidime, carbenicillin, and trimethoprim was significantly reduced after biofilm formation. This phenomenon is attributed to the physical barrier of the biofilm, which impedes antibiotic penetration, and the diminished metabolic activity of bacteria within the biofilm. When L2 is combined with antibiotics such as amikacin, the sensitivity of *Pseudomonas aeruginosa* biofilm bacteria to these antibiotics can be significantly enhanced. These findings suggest that the clinical application of L2 in combination with antibiotics may improve the therapeutic effect [[46], [47], [48]].

In summary, this study not only rapidly identified the candidate QSI L2 from a compound library via an integrated computational and experimental strategy but also assessed its effectiveness as a monotherapy both in vitro and in vivo. Furthermore, L2 has been demonstrated to exhibit synergistic effects when used in combination with antibiotics, significantly enhancing the susceptibility of *P. aeruginosa* biofilm bacteria to antibiotics. Overall, these findings underscore the potential of L2 as a promising QSI worthy of further development for treating *P. aeruginosa* infections.

## CRediT authorship contribution statement

**Juanyan Liu:** Writing – original draft, Methodology, Investigation, Data curation, Conceptualization. **Yi Deng:** Investigation. **Xinran Wang:** Investigation. **Piao Luo:** Investigation. **Shuaiwen Ding:** Investigation. **Weizhong Zhang:** Investigation. **Shahab Uddin:** Investigation. **Hongyu Li:** Writing – review & editing, Supervision, Resources, Conceptualization. **Yang Li:** Writing – review & editing, Supervision, Resources, Funding acquisition, Conceptualization.

## Ethics approval

Not required.

## Funding

**Table:** 

Funder	Grant(s)	Author(s)
National Natural Science Foundation of China	No.31571989, No.31772147	Hongyu Li
Major Science and Technology Project of Gansu Province	No.23ZDFA013-4, No.31571989	Yang Li

## Declaration of competing interest

The authors declare that they have no known competing financial interests or personal relationships that could have appeared to influence the work reported in this paper.

## Data Availability

Data will be made available on request.
