# Supplementary material for: Novel PHL derivatives targeting quorum sensing: A strategy to combat Pseudomonas aeruginosa infections
Source: Biofilm. 2025 Oct 24;10:100326. doi: 10.1016/j.bioflm.2025.100326 (PMC12718213; doi:10.1016/j.bioflm.2025.100326)
Supplement: Multimedia component 1 [file mmc1.docx]

**Supplementary Tables**

**Table S1. 1H NMR and 13C NMR data of PHL derivatives.**

| **Compounds** | **Traits and yield** | | **1H NMR and 13C NMR** |
| --- | --- | --- | --- |
| L1 | | Yellow solid; 70.23% | 1H NMR (400 MHz, DMSO) δ 8.05, 8.03, 7.46, 7.43, 2.88, 2.86, 2.84, 2.73, 2.61, 2.59, 2.57.13C NMR (100 MHz, DMSO) δ 171.16, 150.72, 146.27, 130.17, 123.69, 38.65, 36.98, 35.28, 33.67, 30.81. |
| L2 | | Yellow solid; 69.45% | 1H NMR (400 MHz, DMSO) δ 8.38, 8.36, 8.17, 8.15, 7.51, 7.49, 4.60, 4.57, 4.57, 4.55, 4.55, 4.53, 4.39, 4.38, 4.37, 4.36, 4.35, 4.34, 4.26, 4.24, 4.24, 4.23, 4.21, 4.19, 2.76, 2.74, 2.72, 2.44, 2.44, 2.43, 2.42, 2.42, 2.41, 2.41, 2.41, 2.40, 2.40, 2.39, 2.39, 2.38, 2.37, 2.23, 2.21, 2.20, 2.19, 2.17, 2.15, 1.90, 1.89, 1.87, 1.85, 1.83. 13C NMR (100 MHz, DMSO) δ 175.86, 172.23, 150.70, 146.34, 130.11, 123.89, 65.73, 48.35, 34.68, 34.58, 28.64, 26.77. |
| L6 | | Yellow solid; yield: 73.97% | 1H NMR (400 MHz, DMSO) δ 8.34, 8.32, 7.64, 7.62, 7.03, 7.01, 4.57, 4.54, 4.52, 4.50, 4.37, 4.34, 4.32, 4.23, 4.21, 4.19, 4.17, 2.55, 2.53, 2.51, 2.42, 2.40, 2.37, 2.35, 1.82, 1.80, 1.78, 1.76, 1.74.13C NMR (100 MHz, DMSO) δ 175.85, 172.33, 141.99, 137.46, 131.37, 91.77, 65.71, 48.32, 34.75, 34.27, 28.67, 27.07. |
| L9 | | Yellow solid; yield: 75.76% | 1H NMR (400 MHz, DMSO) δ 7.29, 7.28, 7.27, 7.26, 7.10, 7.08, 7.06, 2.92, 2.82, 2.80, 2.78, 2.60, 2.58, 2.56.13C NMR (101 MHz, DMSO) δ 171.55, 162.31, 159.92, 138.15, 130.62, 130.54, 115.36, 115.15, 37.01, 35.25, 34.63, 30.23. |
| L10 | | Yellow solid; yield: 73.82% | 1H NMR (400 MHz, DMSO) δ 7.25, 7.23, 7.22, 7.21, 7.12, 7.10, 7.07, 2.92, 2.81, 2.60, 2.58, 2.56, 2.30, 2.28, 2.26, 1.81, 1.79, 1.77, 1.75, 1.74.13C NMR (100 MHz, DMSO) δ 172.01, 162.25, 159.86, 138.49, 130.46, 130.38, 115.45, 115.24, 37.05, 35.19, 34.25, 32.10, 27.05. |

**^1^H NMR spectrum of the compound L1**

**^13^C NMR spectrum of the compound L1**

**^1^H NMR spectrum of the compound L2**

**^13^C NMR spectrum of the compound L2**

**^1^H NMR spectrum of the compound L6**

**^13^C NMR spectrum of the compound L6**

**^1^H NMR spectrum of the compound L9**

**^13^C NMR spectrum of the compound L9**

**^1^H NMR spectrum of the compound L10**

**^13^C NMR spectrum of the compound L10**

**Table S2. Determination of MIC of PAO1 planktonic and biofilm bacteria by different antibiotics.**

| **Antibiotics** | **MIC of *P. aeruginosa* PAO1 planktonic bacteria (μg/mL)** | **MIC of *P. aeruginosa* PAO1 biofilm bacteria (μg/mL)** |
| --- | --- | --- |
| Amikacin | 2.5 | 5 |
| Ceftazidime | 1.25 | 40 |
| Ciprofloxacin | 0.16 | 0.16 |
| Carbenicillin | 2.5 | 5 |
| Trimethoprim | 1.25 | 10 |

**Table S3. Primers used in this study.**

| **Primer name** | **Genes** | **Oligonucleotide sequences (5′→3′)** | **Tm (°C)** | **GC%** |
| --- | --- | --- | --- | --- |
| *lasR*-F | *lasR* | ACGCTCAAGTGGAAAATTGG | 60.11 | 45.00 |
| *lasR*-R | *lasR* | GTAGATGGACGGTTCCCAGA | 59.93 | 55.00 |
| *lasI*-F | *lasI* | CTACAGCCTGCAGAACGACA | 60.20 | 55.00 |
| *lasI*-R | *lasI* | ATCTGGGTCTTGGCATTGAG | 60.07 | 50.00 |
| *rhlR*-F | *rhlR* | AGGAATGACGGAGGCTTTTT | 60.07 | 45.00 |
| *rhlR*-R | *rhlR* | CCCGTAGTTCTGCATCTGGT | 60.13 | 55.00 |
| *rhlI*- F | *rhlI* | CTCTCTGAATCGCTGGAAGG | 60.09 | 55.00 |
| *rhlI*- R | *rhlI* | GACGTCCTTGAGCAGGTAGG | 59.87 | 60.00 |
| *mvfR*-F | *mvfR* | AACCTGGAAATCGACCTGTG | 59.97 | 50.00 |
| *mvfR*-R | *mvfR* | TGAAATCGTCGAGCAGTACG | 60.01 | 50.00 |
| *16srRNA*-F | *16srRNA* | GCGCAACCCTTGTCCTTAGTT | 57.80 | 52.38 |
| *16srRNA*-R | *16srRNA* | TGTCACCGGCAGTCTCCTTAG | 59.70 | 57.14 |

F=Forward primer, R=Reverse primer

**Table S4. Drug resistance information for clinical strains of *P. aeruginosa*.**

**Table S4.1. Drug resistance information of *Pseudomonas aeruginosa* clinical strain C1.**

| **Antimicrobia** | **MIC(μg/mL)** | **Explanation of results** | **Folding point criteria** | | **Antimicrobia** | **MIC(μg/mL)** | **Explanation of results** | **Folding point criteria** | |
| --- | --- | --- | --- | --- | --- | --- | --- | --- | --- |
|  |  |  | **S** | **R** |  |  |  | **S** | **R** |
| Colistin | ≤1 |  | ≤2 | ≥4 | Aztreonam | 32 | R | ≤8 | ≥32 |
| Levofloxacin | >8 | R | ≤1 | ≥4 | Cefepime | 8 | S | ≤8 | ≥32 |
| Ciprofloxacin | >4 | R | ≤0.5 | ≥2 | Piperacillin | 16\4 | S | ≤16 | ≥128 |
| Amikacin | ≤8 | S | ≤16 | ≥64 | Tobramycin | >8 | R | ≤4 | ≥16 |
| Meropenem | 2 | S | ≤2 | ≥8 | Gentamicin | >8 | R | ≤4 | ≥16 |
| Imipenem | 1.5 | S | ≤2 | ≥8 | Ceftazidime | 8 | S | ≤8 | ≥32 |

R= Resistant, S=Sensitive, I=Intermediate.

**Table S4.2. Drug resistance information of *P. aeruginosa* clinical strain C2.**

| **Antimicrobia** | **MIC(μg/mL)** | **Explanation of results** | **Folding point criteria** | | **Antimicrobia** | **MIC(μg/mL)** | **Explanation of results** | **Folding point criteria** | |
| --- | --- | --- | --- | --- | --- | --- | --- | --- | --- |
|  |  |  | **S** | **R** |  |  |  | **S** | **R** |
| Colistin | ≤1 |  | ≤2 | ≥4 | Aztreonam | 32 | R | ≤8 | ≥32 |
| Levofloxacin | 32\8 |  |  |  | Cefepime | 8 | S | ≤8 | ≥32 |
| Ciprofloxacin | 4 | R | ≤1 | ≥4 | Piperacillin | 16\4 | S | ≤16 | ≥128 |
| Amikacin | 1 | I | ≤0.5 | ≥2 | Tobramycin | ≤2 | S | ≤4 | ≥16 |
| Meropenem | ≤8 | S | ≤16 | ≥64 | Gentamicin | ≤2 | S | ≤4 | ≥16 |
| Imipenem | 1 | S | ≤2 | ≥8 | Ceftazidime | 8 | S | ≤8 | ≥32 |

R= Resistant, S=Sensitive, I=Intermediate.

**Table S4.3. Drug resistance information of *P. aeruginosa* clinical strain C3.**

| **Antimicrobia** | **MIC(μg/mL)** | **Explanation of results** | **Folding point criteria** | | **Antimicrobia** | **MIC(μg/mL)** | **Explanation of results** | **Folding point criteria** | |
| --- | --- | --- | --- | --- | --- | --- | --- | --- | --- |
|  |  |  | **S** | **R** |  |  |  | **S** | **R** |
| Colistin | ≤1 |  | ≤2 | ≥4 | Aztreonam | >32 | R | ≤8 | ≥32 |
| Levofloxacin | >8 | R | ≤1 | ≥4 | Cefepime | ≤1 | S | ≤8 | ≥32 |
| Ciprofloxacin | >4 | R | ≤0.5 | ≥2 | Piperacillin | ≤4/4 | S | ≤16 | ≥128 |
| Amikacin | ≤8 | S | ≤16 | ≥64 | Tobramycin | >8 | R | ≤4 | ≥16 |
| Meropenem | >8 | R | ≤2 | ≥8 | Gentamicin | >8 | R | ≤4 | ≥16 |
| Imipenem | >8 | R | ≤2 | ≥8 | Ceftazidime | >32 | R | ≤8 | ≥32 |

R= Resistant, S=Sensitive, I=Intermediate.

**Table S5.** Combinational activities of L2 and antibiotics against *Pseudomonas aeruginosa*.

**Table S5.1.** Combinational activities of L2 and Ceftazidime against *Pseudomonas aeruginosa*.

| Concentration of L2 (μM) | MIC of Ceftazidime (μg/mL) | FIC of Ceftazidime | FIC of L2* | FICI | Interpretation |
| --- | --- | --- | --- | --- | --- |
| 0 | 40 | - | - | - | - |
| 12.5 | 8 | 0.2 | 0.016 | 0.216 | Synergy |
| 25 | 3 | 0.075 | 0.031 | 0.106 | Synergy |
| 50 | 9 | 0.225 | 0.063 | 0.288 | Synergy |
| 100 | 8 | 0.2 | 0.125 | 0.325 | Synergy |
| 200 | 9 | 0.225 | 0.25 | 0.475 | Synergy |

Fractional inhibitory concentration index (FICI), which depends on the fractional inhibitory concentration (FIC) of each compound, was interpreted below as follows: FICI≤0.5, synergy; 0.5 < FICI ≤1, additive effect; 1 < FICI≤4, no interaction; FICI >4, antagonism

^*^The MIC of L2 alone was set to 800 μM (>400 μM) for FICI calculation.

**Table S5.2.** Combinational activities of L2 and Amikacin against *Pseudomonas aeruginosa.*

| Concentration of L2 (μM) | MIC of Amikacin (μg/mL) | FIC of Amikacin | FIC of L2^*^ | FICI | Interpretation |
| --- | --- | --- | --- | --- | --- |
| 0 | 5 | - | - | - | - |
| 12.5 | 1.25 | 0.25 | 0.016 | 0.266 | Synergy |
| 25 | 0.325 | 0.065 | 0.031 | 0.096 | Synergy |
| 50 | 1.25 | 0.25 | 0.063 | 0.313 | Synergy |
| 100 | 1.3 | 0.26 | 0.125 | 0.385 | Synergy |
| 200 | 1.25 | 0.25 | 0.25 | 0.5 | Synergy |

Fractional inhibitory concentration index (FICI), which depends on the fractional inhibitory concentration (FIC) of each compound, was interpreted below as follows: FICI≤0.5, synergy; 0.5 < FICI ≤1, additive effect; 1 < FICI≤4, no interaction; FICI >4, antagonism

^*^The MIC of L2 alone was set to 800 μM (>400 μM) for FICI calculation.

**Table S5.3.** Combinational activities of L2 and Carbenicillin against *Pseudomonas aeruginosa*.

| Concentration of L2 (μM) | MIC of Carbenicillin  (μg/mL) | FIC of Carbenicillin | FIC of L2^*^ | FICI | Interpretation |
| --- | --- | --- | --- | --- | --- |
| 0 | 5 | - | - | - | - |
| 12.5 | 2.2 | 0.44 | 0.016 | 0.456 | synergy |
| 25 | 2 | 0.4 | 0.031 | 0.431 | synergy |
| 50 | 2 | 0.4 | 0.063 | 0.463 | synergy |
| 100 | 2 | 0.4 | 0.125 | 0.525 | additive effect |
| 200 | 3 | 0.5 | 0.25 | 0.75 | additive effect |

Fractional inhibitory concentration index (FICI), which depends on the fractional inhibitory concentration (FIC) of each compound, was interpreted below as follows: FICI≤0.5, synergy; 0.5 < FICI ≤1, additive effect; 1 < FICI≤4, no interaction; FICI >4, antagonism

^*^The MIC of L2 alone was set to 800 μM (>400 μM) for FICI calculation.

**Table S5.4.** Combinational activities of L2 and Trimethoprim against *Pseudomonas aeruginosa*.

| Concentration of L2 (μM) | MIC of Trimethoprim  (μg/mL) | FIC of Trimethoprim | FIC of L2* | FICI | Interpretation |
| --- | --- | --- | --- | --- | --- |
| 0 | 10 | - | - | - | - |
| 12.5 | 3 | 0.3 | 0.016 | 0.316 | synergy |
| 25 | 1.2 | 0.12 | 0.031 | 0.151 | synergy |
| 50 | 3 | 0.3 | 0.063 | 0.363 | synergy |
| 100 | 2.5 | 0.25 | 0.125 | 0.375 | synergy |
| 200 | 2.5 | 0.25 | 0.25 | 0.5 | synergy |

Fractional inhibitory concentration index (FICI), which depends on the fractional inhibitory concentration (FIC) of each compound, was interpreted below as follows: FICI≤0.5, synergy; 0.5 < FICI ≤1, additive effect; 1 < FICI≤4, no interaction; FICI >4, antagonism

^*^The MIC of L2 alone was set to 800 μM (>400 μM) for FICI calculation.

**‘**

**Supplementary Methods
 Chemical Synthesis**


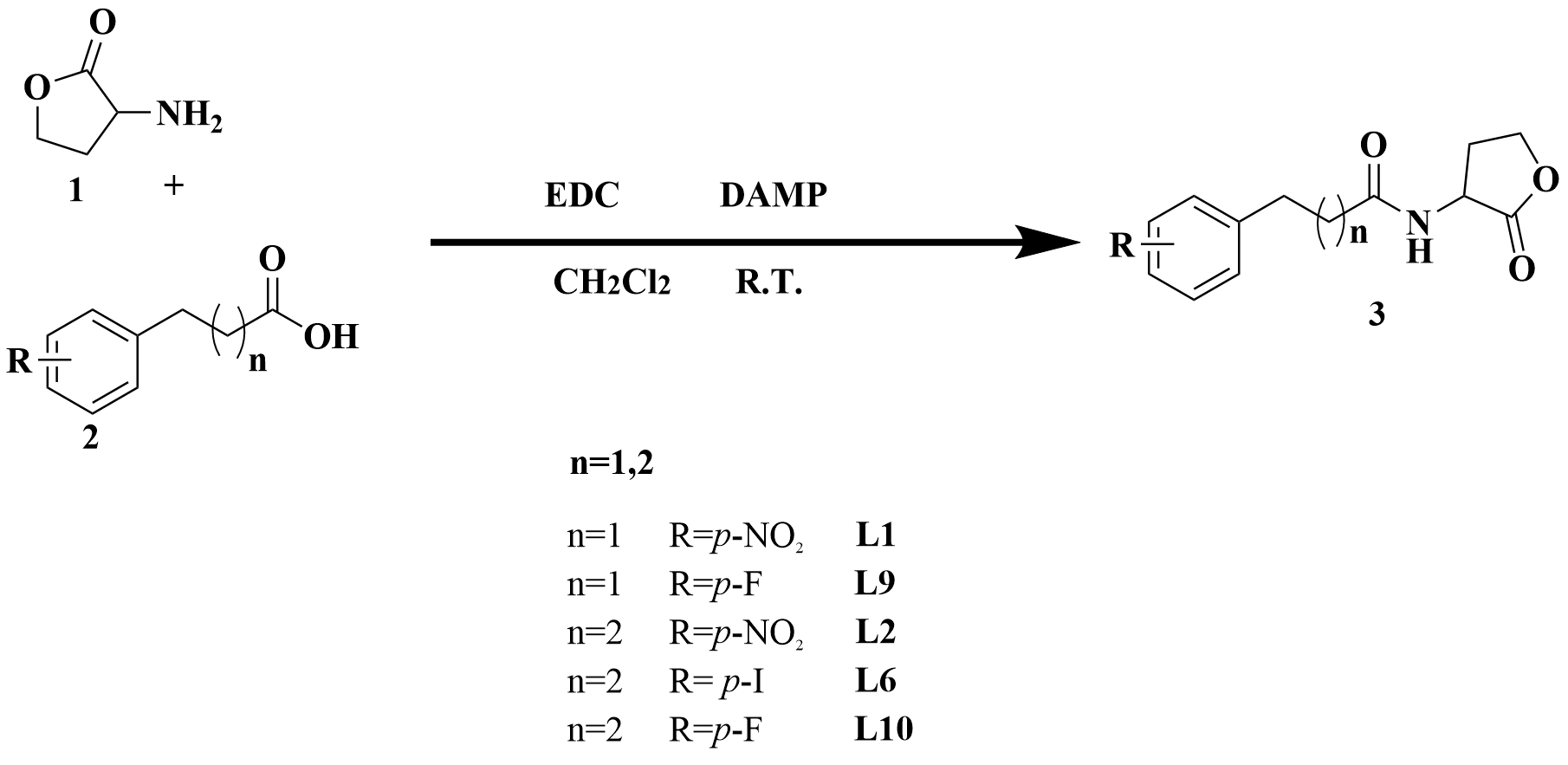


The derivatives of carboxylic acids and amines were mixed at a ratio of 1:1 and dissolved in dichloromethane (DCM), then 2.5 eq N, N – Diisopropylethylamine (DIEPA) and 1.0 eq O-(7 - Azabenzotriazol - 1 - yl)-N, N, N’, N'-tetramethyluronium hexafluorophosphate **(**HATU) were added to the mixture. The reaction mixture was stirred at room temperature for 18-24 h. After the reaction, the above reaction solution was washed three times with an equal volume of 10% HCl solution and saturated NaCl solution. The organic phase was collected and dried with anhydrous Na_2_SO4. The crude product was purified by silica gel column chromatography to obtain the target compounds **L1**, **L2**, **L6**, **L9,** and **L10**.

**FICI Calculation Method**

The Fractional Inhibitory Concentration Index (FICI) was calculated as follows: FICI = FIC_ antibiotic + FIC_L2 = (MIC of antibiotic in combination / MIC of antibiotic alone) + (concentration of L2 in combination / MIC of L2 alone). Since L2 exhibited no intrinsic antimicrobial activity at the highest concentration tested (400 μM), its MIC was set at 800μM for FICI calculations, as commonly described in previous studies for evaluating synergistic agents(1-3).

**Supplementary Figures**

**Figure S1.** **Effects of L2 at various concentrations on the growth of *P. aeruginosa.***

1. Zhou H, Wang W, Cai L, Yang T. Potentiation and Mechanism of Berberine as an Antibiotic Adjuvant Against Multidrug-Resistant Bacteria. Infect Drug Resist. 2023;16:7313-26.

2. Zhong Y, Chen F, Chen D, He Q, Zhang X, Lan L, et al. Design, synthesis, and optimization of TarO inhibitors as multifunctional antibiotics against Methicillin-resistant Staphylococcus aureus. NPJ Antimicrob Resist. 2025;3:28.

3. Scandorieiro S, Rodrigues BCD, Nishio EK, Panagio LA, de Oliveira AG, Durán N, et al. Biogenic Silver Nanoparticles Strategically Combined With Origanum vulgare Derivatives: Antibacterial Mechanism of Action and Effect on Multidrug-Resistant Strains. Frontiers in Microbiology. 2022; Volume 13 - 2022.
